# Efficient Production and Purification of Recombinant Murine Kindlin-3 from Insect Cells for Biophysical Studies

**DOI:** 10.3791/51206

**Published:** 2014-03-19

**Authors:** Luke A. Yates, Robert J. C. Gilbert

**Affiliations:** ^1^Division of Structural Biology, Wellcome Trust Centre for Human Genetics, University of Oxford

**Keywords:** Virology, Issue 85, Heterologous protein expression, insect cells, *Spodoptera frugiperda*, baculovirus, protein purification, kindlin, cell adhesion

## Abstract

Kindlins are essential coactivators, with talin, of the cell surface receptors integrins and also participate in integrin outside-in signalling, and the control of gene transcription in the cell nucleus. The kindlins are ~75 kDa multidomain proteins and bind to an NPxY motif and upstream T/S cluster of the integrin β-subunit cytoplasmic tail. The hematopoietically-important kindlin isoform, kindlin-3, is critical for platelet aggregation during thrombus formation, leukocyte rolling in response to infection and inflammation and osteoclast podocyte formation in bone resorption. Kindlin-3's role in these processes has resulted in extensive cellular and physiological studies. However, there is a need for an efficient method of acquiring high quality milligram quantities of the protein for further studies. We have developed a protocol, here described, for the efficient expression and purification of recombinant murine kindlin-3 by use of a baculovirus-driven expression system in Sf9 cells yielding sufficient amounts of high purity full-length protein to allow its biophysical characterization. The same approach could be taken in the study of the other mammalian kindlin isoforms.

**Figure:**
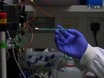


## Introduction

Proteins of the kindlin family are a crucial component of focal adhesion assembly, and therefore essential for complex life. Kindlins, of which there are 3 isoforms in mammals (kindlin-1, kindlin-2, and kindlin-3), are considered coactivators of the extracellular receptors integrins alongside talin^1^. Integrin-mediated cell adhesion connects the cell surface to the extracellular matrix (ECM) in higher eukaryotes. It is a critical and common process in a plethora of physiological phenomena, which include tissue integrity, embryogenesis, bone metabolism, hemostasis, and immunity. Integrin-mediated cell adhesion is activated through inside-out signal transduction via the binding of talin and kindlin to the integrin β-subunit cytoplasmic tails (CTs) at their conserved NPxY motifs. The biomedical importance of kindlin proteins extends however as far as the nucleus, where kindlin-2 has been shown in several recent reports to be involved in transcriptional control^2,3^.

Kindlins are multidomain proteins of approximately 75 kDa, hallmarked by the possession of a bipartite C-terminal FERM (4.1 band, ezrin, radixin, moesin) domain, which is interrupted by a pleckstrin homology (PH) domain at the center of its F2 subdomain^4,5^. Studies of the kindlin-2 and kindlin-3 PH domains showed that it binds to the lipid second messengers phosphatidylinositol-(3,4,5)-trisphosphate and phosphatidylinositol-(4,5)-bisphosphate^6-8^. However, studies of the kindlin-1 PH domain show that it binds to PtdIns(3,4,5)P_3 _with a much lower affinity, which can be explained in kindlin-1 by an isoform-specific salt bridge preventing lipids from binding^9^. In addition, there is an ~100 amino acid loop inserted into the F1 domain of the kindlins that is predicted to be unfolded but binds to phosphatidylserine in the inner leaflet of the plasma membrane^10,11^. The kindlin FERM domain is considered homologous to the talin FERM domain, although the talin FERM domain does not possess a pleckstrin homology domain. Both kindlins and talin interact with NPxY motifs on the integrin β-tails via the F3 region of their FERM domain, but kindlin binds to the membrane distal motif, while talin targets the membrane proximal one^12-16^. Kindlins and talin both in addition possess an N-terminal F0 domain with a ubiquitin-like fold that is not found in other FERM proteins^11,17^. Studies on the F0 domain of kindlin-2 have shown that it independently binds to phosphatidylinositol-(4,5)-bisphosphate enriched membranes^17^.

The kindlins exhibit paralogue-specific tissue expression patterns and nonredundant physiological functions. Kindlin-1 is primarily expressed in the epidermis, but also to a lesser extent the colon, stomach, and kidneys; kindlin-2 is ubiquitously expressed but is concentrated in striated and smooth muscle and is the only kindlin expressed in embryonic development^4^; and kindlin-3 is expressed in the hematopoietic tissues with the highest concentration of kindlin-3 found in megakaryocytes^18^. However, more recent studies have suggested that functional protein is expressed in endothelial tissues as well^19^.

Kindlin-3 is of acute medical interest due to its important physiological role in the blood. It is critical for platelet aggregation and spreading during thrombus formation^20^, leukocyte rolling in response to infection and inflammation^21,22^ and osteoclast podocyte formation in bone resorption^23^. Furthermore, depletion of kindlin-3 in humans leads to leukocyte adhesion deficiency type-III - a disease characterized by life-threatening bleeding disorders and recurrent bacterial infections^20,24,25^. Kindlin-3 knock-out studies in mice revealed the crucial function of the protein in cell adhesion. *KIND3*^-/-^ mice display distinct phenotypes, such as severe bleeding due to inactive platelet integrins, severe osteopetrosis, and impaired leukocyte adhesion^20,22^, resembling symptoms in humans lacking kindlin-3.

High-resolution structural data on the kindlins, to date, has been restricted to individual subdomains such as the pleckstrin homology (PH) domain of kindlin-1^9^ and kindlin-2^26,27 ^and the F0 domain of kindlin-1^11^ and kindlin-2^17^. Most of the subdomains of each kindlin polypeptide have however resisted cloning and structural analysis (Yates and Gilbert, unpublished observations), and studies of the full-length proteins have been hindered by the difficulty of expressing and purifying sufficient quantities using *E. coli* (unpublished observations and Harburger* et al.*^14^). There is considerable medical interest in kindlin-3 and its function, alongside the other two family members, and recently we generated milligram quantities of it by recombinant expression in *Spodoptera frugiperda *cells driven by baculovirus infection^12^. We therefore here describe methods for the production of milligram quantities of recombinant mouse kindlin-3 in insect cell culture, suitable for extensive structural studies and biochemical analysis.

In this protocol we make use of an engineered knockout bacmid (BAC10_KO:1629_)that is, alone, unable to produce viable virions^28^. The viral DNA is thus rescued by recombination with a transfer vector that in this case also includes the kindlin-3 gene (*FERMT3*) and results in the *FERMT3* gene replacing the virus very late gene, which is highly expressed but redundant, resulting in a recombinant virus that expresses mouse kindlin-3 as part of the virus life cycle^28^.We identified this method for the production of kindlin-3 after attempts to express and purify it in other expression hosts proved prohibitively difficult (unpublished observations) but also due to the versatility of the pOPIN vector suite, which we used for cloning and that can deployed in many expression hosts^29^.

## Protocol

This protocol assumes that the mouse kindlin-3 gene (*FERMT3*) has been successfully cloned into a vector downstream of the very late p10 promoter and that the vector possesses flanking baculovirus sequences to permit recombination with the bacmid BAC10_KO:1629_ developed by I.M. Jones and colleagues^28^. For this protocol, the kindlin-3 gene was cloned into pOPINE^29^ and the primers and cloning strategy used can be found described elsewhere^12^. The plasmid is engineered so that the *FERMT3 *gene (kindlin-3) is under the control of the p10 baculoviral promoter and the vector contains 5′ UTR/ORF603 and ORF 1629 and encodes a C-terminal His_6_-tag for downstream purification^29^.

### 1. Insect Cell Culture and Maintenance

1. Prior to recombinant baculovirus amplification, *Spodoptera frugiperda* cells adapted to suspension culture (Sf9 cells) should be grown and maintained.****Insect cells should always be handled using aseptic techniques in a dedicated tissue culture fume hood.
2. Suspension cultures of insect cells are incubated in Sf-900 II (SFM) serum free liquid medium supplemented with 100 μg/ml penicillin and 100 μg/ml streptomycin in flasks at 27 °C with shaking at 100 rpm.
3. Maintain the insect cell culture density range between 1 x 10^6 ^- 1 x 10^7 ^cells/ml by splitting and diluting the cell culture with fresh Sf-900 II media. Note: healthy****cells should look uniform in size and should be spherical in shape.
4. Count the cells in a sample volume using a hemocytometer and light microscopy to calculate the culture cell density.

### 2. Generation of Recombinant Baculovirus

1. Culture and maintain Sf9 cells in suspension using Sf-900 II media supplemented with 100 μg/ml penicillin and 100 μg/ml streptomycin. For baculovirus generation, insect cells should be cultured to a density range of 5 x 10^5^ - 1 x 10^6^ cells/ml.
2. Seed approximately 1 x 10^6^ Sf9 cells per well of a sterile 6-well tissue culture plate in 2 ml of Sf-900 II media supplemented with 100 μg/ml penicillin and 100 μg/ml streptomycin. Leave the Sf9 cells at RT in the fume hood to adhere to the base of the plastic wells and thus form a monolayer.
3. Perform recombinant baculovirus generation by cotransfecting bacmid DNA and plasmid DNA onto monolayer culture.
  1. For each transfection, mix 1-2 μg of purified pOPINE-*mFERMT3,* which possesses the ORF1629 baculovirus elements, with 0.5 μg of purified BAC10_KO:1629_ in 100 μl of Sf-900 II SFM without antibiotics (Solution A).
  2. In a separate tube, dilute 6 μl of Cellfectin II Reagent with 100 μl of Sf-900 II SFM without antibiotics for each transfection reaction (Solution B). A ‘master mix' can be created here, for reduced liquid handling, if many transfections are required.
  3. Mix the two solutions (A and B, approximately 200 μl) and incubate at RT for 20 min to form a lipid-DNA complex.
  4. Dilute the lipid-DNA complexes with 800 μl Sf-900 II SFM without antibiotics. Carefully aspirate the Sf9 cell monolayer media and carefully pipette the solution A/B and media on top of the Sf9 monolayer.
  5. Incubate the transfected cells in a humidified incubator at 27 °C O/N and add a further 1 ml of Sf-900 II SFM without antibiotics to each monolayer culture the following day. Incubate cells at 27 °C for a further 5 days.
4. Harvest the recombinant baculovirus directly from the culture medium (approximately 2 ml in total) and transfer to a clean centrifuge tube (for example a 15 ml Falcon tube).
  1. Clarify any Sf9 cell debris by centrifugation at 1,000 x g for 5 min at RT. Transfer the virus, which is in the resulting supernatant and denoted P1, to a clean tube and store at 4 °C in the dark until use. At this stage the remaining Sf9 monolayer can be used to assess recombinant virus generation by assessing the presence of recombinant kindlin-3 in the insect cells.
5. Resuspend the monolayer with 0.5 ml PBS and dilute a 10 μl sample with equal volumes of 2x SDS-PAGE loading buffer. Heat the samples at >95 °C for at least 10 min.
  1. Sonicate the sample for 1 sec at 10% amplitude using a micro-sonicator tip if it is too viscous for proper gel loading.

### 3. Amplification of Recombinant Baculovirus

1. For the amplification of virus in suspension, use a cell density of 1.4 x 10^6^ cells/ml and this should occupy 1/20 of the total flask volume (*i.e.* 50 ml culture in a 2 L flask).
2. Achieve the amplification of the recombinant virus by infecting the insect cell culture with the P1 viral stock at a multiplicity of infection (MOI) of 0.1 using the following formula; 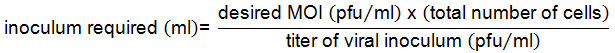 Note: P1 virus generation can be assumed to have an expected viral titer of 1 x 10^7^ pfu/ml. However, a plaque assay can be performed prior to this stage.
3. Incubate the P1-infected insect culture at 27 °C with shaking at 100 rpm for 3 days (72 hr).
4. Harvest the virus by separating the cells from the media by centrifugation at 1,000 x g for 5 min at RT. Save the resulting cell pellet at this stage for confirmation of recombinant virus production by assessing kindlin-3 protein expression by SDS-PAGE and western blotting.
5. Transfer the clarified virus-enriched media to a clean tube and store at 4 °C in the dark until use. This viral stock is denoted as P2. Note: A viral titer of 2 x 10^8^ pfu/ml (or an amplification rate of 100 pfu/cell) can be anticipated.

### 4. Expression of kindlin-3 in Baculovirus-Infected Sf9

1. Grow an adequate volume of Sf9 cells cultures in suspension in Sf-900 II SFM supplemented with 100 μg/ml penicillin and 100 μg/ml streptomycin, prior to large-scale recombinant kindlin-3 expression for purification. Incubate the suspension cultures at 27 °C with shaking at 100 rpm and with a total culture volume:flask volume ratio of 1:5.
2. Infect suspension cultures of Sf9 at a density of 2 x 10^6^ cells/ml.
3. Supplement Sf9 cultures with a final concentration of 1% (v/v) Fetal Bovine Serum (FBS) followed with amplified recombinant virus (P2-viral stock) to give a MOI of 1. Incubate the infected cultures at 27 °C with shaking at 100 rpm.
4. Harvest recombinant kindlin-3-CHIS_6_-expressing Sf9 cells 72 hr post-infection by centrifugation at 1,000 x g* *and the resulting cell pellet stored at -20 °C until use, or -80 °C for long term storage.

### 5. Purification of Recombinant Kindlin-3

1. Thaw frozen baculovirus-infected insect cell (Sf9) pellets expressing recombinant kindlin-3 on ice.
2. Resuspend the thawed cell pellet with lysis buffer (50 mM Tris-HCl, pH 7.5, 500 mM NaCl, 1% (v/v) Tween-20), supplemented with EDTA-free protease inhibitor cocktail and 1,000-2,000 U of DNase1. Note: Alternatively, modified phosphate buffered saline (PBS) can also be used, where the NaCl concentration is adjusted to 500 mM to prevent nonspecific interactions between endogenous Sf9 cell proteins and the immobilized metal affinity column used for downstream purification (see below).
3. Lyse the cells by incubating the resuspended cells with the detergent and vortexing. Sonicate (40% amplitude, 10 cycles of 10 sec pulse followed by 10 sec cooling) the sample for further cell disruption in an ice bath or alternatively use a Dounce homogenizer.
4. Clarify the lysate by centrifugation at 48,000 x g for 1 hr at 4 °C. Load the resulting supernatant onto a HisTrap column (5 ml column volume), pre-equilibrated with lysis buffer, at 4 °C at a rate of 1 ml/min. Note: Alternatively, the clarified lysate can be incubated with a 1-5 ml bed volume of pre-equilibrated nickel sepharose beads (*e.g.* Ni Sepharose 6 fast flow) at 4 °C for 1-2 hr. A column of Ni sepharose can be formed after the binding step by using a gravity flow column.
5. Wash the column with 10 column volumes of wash buffer (50 mM Tris-HCl, pH 7.5, 500 mM NaCl, 10 mM imidazole) to remove unbound proteins.
6. Use a linear imidazole gradient from 10-500 mM at a rate of 10 mM/ml (or in 10 column volumes) using an Äkta FPLC to elute the bound recombinant kindlin-3-CHIS_6_.
7. Fractionate the elution into 0.5-1 ml fractions using the Äkta FPLC, with those fractions containing kindlin-3-CHIS_6_ usually eluting at an imidazole concentration of 300 mM.
8. Assess the protein composition of the eluant by SDS-PAGE and for first-time purifications confirm by western blotting using an anti-His_6 _antibody or anti-mouse kindlin-3 antibody.
9. Pool the fractions containing kindlin-3 and buffer exchange into 20 mM Tris-HCl, pH 7.5, 200 mM NaCl via a series of dilutions into buffer and sample concentrations using a centrifugal protein concentrator with a 50 kDa molecular weight cut-off (MWCO) at 4 °C. Note: Alternatively, dialyze the protein solution using Slide-A-lyzer Dialysis Cassette with a MWCO of 30 kDa at 4 °C for 4 hr to O/N into 20 mM Tris-HCl, pH 7.5, 200 mM NaCl. Note: for ion exchange (see below) a lower concentration of NaCl, *i.e.* 50 mM, can be and has been used for this step.
10. Apply the buffer-exchanged protein solution onto a pre-equilibrated HiTrap heparin HP column (5 ml column volume) using an Äkta FPLC at a rate of 0.5 ml/min. Note: The bound kindlin-3 is eluted using a linear NaCl gradient (0.2 M NaCl to 1 M NaCl) in the same buffer, increasing at a rate of 10 mM/ml. Kindlin-3-CHIS6 is expected to elute at ~0.6 M NaCl.
11. Fractionate the elution into 0.5-1 ml fractions and assess the protein composition by SDS-PAGE and western blotting, if appropriate. Note: one can expect a protein purity of close to 95%, as assessed by SDS-PAGE.
12. Pool fractions containing kindlin-3 and concentrate using a centrifuge protein concentrator with a 50 kDa MWCO to a final volume of 0.5-2 ml.
13. In a final step, polish the concentrated protein and buffer exchange the protein using size exclusion chromatography (SEC).
  1. Apply the purified protein onto a Superdex S200 (16/60) or (10/30) pre-equilibrated in 20 mM Tris-HCl, pH 7.5, 200 mM NaCl, 1 mM DTT at a rate of 1 ml/min or 0.5 ml/min depending on column size used. Note: Size exclusion chromatography can also be performed in phosphate-buffered saline (PBS) if required.
  2. Purify the proteins according to size by applying buffer onto the column at a rate of 1 ml/min or 0.5 ml/min, depending on column size used. Note: The protein eluting from the column is fractionated and monitored using the absorbance at 280 nm.
  3. A single absorbance peak should be expected from SEC, which is fractionated and assessed by SDS-PAGE to determine homogeneity, and is typically >95% pure after this step.
14. Concentrate the purified kindlin-3-CHIS_6_ using a centrifuge protein concentrator with a 50 kDa MWCO to ~15 mg/ml, as assessed spectrophotometrically using a calculated extinction coefficient (ε) of 109,320 M^-1^ cm^-1 ^(assuming all cysteine residues are reduced).
15. For storage at -20 °C and long term storage at -80 °C, aliquot the protein into PCR tubes and flash-freeze the samples in liquid nitrogen. Alternatively, the protein can be used directly for investigation using a number of biochemical and biophysical techniques.

## Representative Results

The large scale expression of recombinant mouse kindlin-3 using baculovirus-infected Sf9 cells can take less than two weeks to achieve milligram quantities, as illustrated in the schematic **Figure 1A** and requires only a small quantity of plasmid DNA from a QIAprep Miniprep kit, for example. The generation of recombinant baculovirus is achieved by cotransfecting Sf9 cells with mouse kindlin-3 (*FERMT3*)-containing plasmid together with an engineered linearized bacmid (BAC10:KO_1629_) and harvesting the newly formed virions after 5-7 days, as shown in **Figure 1A**. This method of baculovirus generation results in 100% recombinant viruses and essentially dispenses the need for plaque purification^28,30^. A representative small-scale (2 ml monolayer) culture will generate a recombinant virus-containing solution with an expected viral titer of 1 x 10^7 ^plaque forming units (pfu) per ml of culture. One can perform a plaque assay to determine the actual viral titer but this is perhaps too labor-intensive when a high number of constructs are tested for structural studies. The success of the virus generation step can be assessed by using an eGFP-containing plasmid in parallel, or, for this kindlin-3 construct, the Sf9 monolayer can be resuspended in PBS and assessed by SDS-PAGE and western blotting, which typically demonstrates a clear band corresponding to a 75 kDa His-tagged protein, as shown in **Figure 1B**. The virus is subsequently amplified to generate sufficient quantities for large-scale (liter volumes) insect cell infection and recombinant protein isolation. The second passage virus (P2) is generated by infecting suspension cultures with an estimated MOI of 0.1 (see protocol). It is vital that the amplification Sf9 suspension culture occupies only a twentieth of the total flask volume. This additional aeration ensures that the resulting virus-containing media, harvested after 3 days (72 hr) post-infection, will generate sufficient quantities of kindlin-3 in the subsequent expression culture. The amplified virus stock (P2) is assumed to possess an expected viral titer of 2 x 10^8 ^pfu/ml based on a conservative estimate of 100 pfu/cell using a cell density of 2 x 10^6 ^cell/ml during the amplification^31^. Generally, for optimum baculovirus amplification and protein production, the Sf9 cells should be uniform in size and spherical, as shown in **Figure 1C**. Additionally, viral amplification and protein expression is maximal at 72 hr post-infection, and both are significantly reduced at 96 hr post-infection.

Once virus has been amplified and used to infect Sf9 cells in large scale experiments recombinant kindlin-3 is partially purified by virtue of its engineered C-terminal His_6_-tag using immobilized metal affinity chromatography, as shown in **Figure 2**. It is important to carry out the purification at 4 °C and that sufficient protease inhibitors have been added to prevent proteolysis. The partially-purified kindlin-3 is further purified to near homogeneity by ion exchange chromatography (IEC) using a heparin column, as shown in **Figure 3**. We employed the use of a heparin column instead of a conventional ionic exchange column, as we predicted that the large number of basic residues, including a poly-lysine stretch within the kindlin-3 F1 domain, would interact strongly with the negatively charged sulphate groups of the column. This strategy is particularly useful for DNA- and RNA-binding proteins with basic nucleic acid-binding patches, for example the RNA-binding terminal uridylyltransferase Cid1^32^. Finally kindlin-3 purity is "polished" by size exclusion chromatography to remove aggregates and achieve homogeneity, as shown in **Figure 4**. The buffers specified in the protocols are standard buffers frequently used in protein purification for structural analysis. Generally, phosphate-based buffers are avoided for the purification of proteins for structural studies, especially crystallization screening due to the formation of phosphate crystals in the crystallization drops (particularly for experiments at 4 °C). However, we performed a Thermofluor-based thermal shift assay to determine which buffers were stabilizing for kindlin-3, as shown in **Figure 5**. Briefly, a purified protein solution is diluted into buffers that cover a range of pH and sodium chloride concentrations, therefore forming a 2-dimensional screen. The melting of the protein is measured by observing the fluorescence from Sypro orange dye (molecular probes), that binds to hydrophobic residues within the folded protein core, over the temperature range 20-95 °C (293-368 K). The temperature mid-point at which the protein unfolds (transition temperature, T_m_) was calculated using the *Opticon Monitor* software and is described elsewhere^33^. Kindlin-3 was observed to be stable at high sodium chloride concentrations (500 mM) within the pH range 7.0-9.0, with a consistent transition temperature (T_m_)of 55 °C. It was also observed that the T_m_ of Kindlin-3 was approximately 55 °C within the pH range 7.0-7.5 irrespective of sodium chloride concentration.

We found that there was limited proteolysis of kindlin-3 during the purification but SDS-PAGE analysis of highly concentrated protein (~15 mg/ml) demonstrated limited contamination with two additional polypeptides of equal intensity. Oddly, however there is no indication of additional species by size exclusion chromatography, as shown in **Figure 4**. It is thus thought that the protein is nicked by proteases but remains folded and hydrodynamically indistinguishable from full-length protein. Interestingly, Calpain is a known protease that cleaves kindlin-3 at Tyrosine373, which is in the β1-β2 loop of the pleckstrin homology (PH) domain^34^ and may explain our observations. Furthermore, western blotting reveals that one of the polypeptide doublets possesses a C-terminal His-tag and the apparent molecular weight of the doublet, when summed, equals 75 kDa, the same molecular weight as the native protein. The yield of purified recombinant kindlin-3 per liter of Sf9 cells (~2 g cell weight) is, at best, 5 mg.


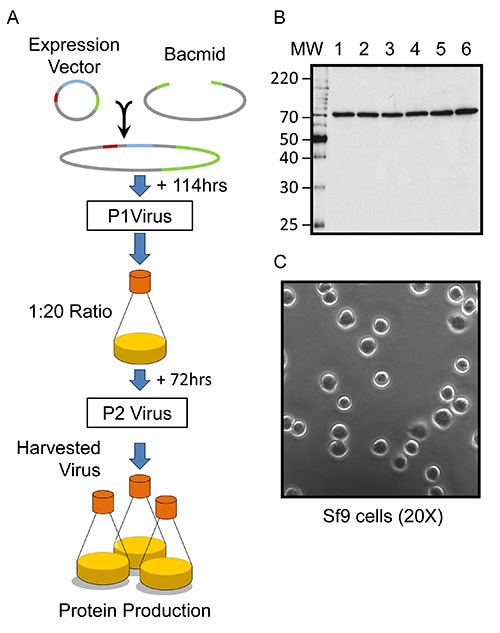
**Figure 1. Overview of baculovirus-infected insect cell heterologous protein expression. **(**A**) A schematized overview of baculovirus generation and expression of kindlin-3 in Sf9 cells. In the DNA vector schematics, 5'UTR/ORF603 is colored in red, the ORF1629 is colored in green and, the gene of the protein of interest (P.O.I.) is colored blue. (**B**)****Western blot, using an anti-His_6 _antibody, of six small-scale (2 ml monolayer) cultures (lanes labelled according to culture) of Sf9 cells producing recombinant baculovirus and recombinant murine kindlin-3.****(**C**)****Light microscopy image of healthy Sf9 cells grown in suspension in Sf-900 II SFM supplemented with antibiotics and transferred to a 35 mm well tissue culture dish (see protocol). Image is 20X magnification.


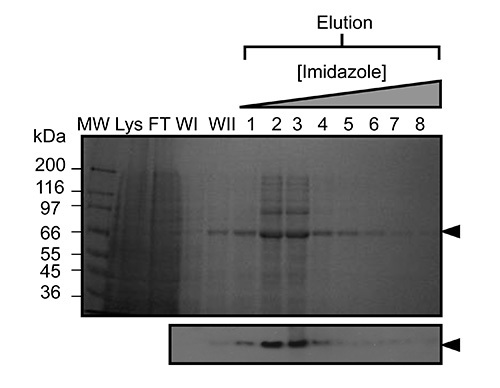
**Figure 2.** **Representative purification of kindlin-3 by immobilized metal affinity chromatography (IMAC).** SDS-PAGE of nickel affinity purified recombinant murine kindlin-3 expressed in baculovirus infected Sf9 cells (~2 g cell weight). Adsorbed protein was eluted using an imidazole gradient (shown above the gel). Lanes are labelled as follows; MW, molecular weight marker; Lys, whole cell lysate; FT, flow through (unbound); WI, wash 1; WII, wash 2. Western blot analysis (below) was also performed using the elution fractions to confirm the presence of the engineered His-tag on the recombinant protein. Please click here to view a larger version of this figure.


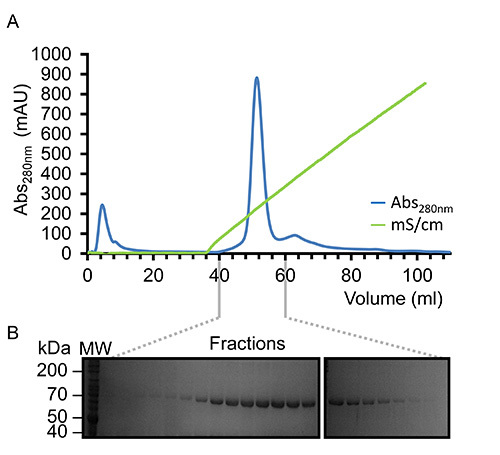
**Figure 3. Representative purification of kindlin-3 by Heparin Affinity Chromatography.** (**A**) Elution profile observed at 280 nm (blue) displaying a single symmetrical peak eluting under a linear sodium chloride gradient (green) using the NaCl concentration range of 0.05-1.0 M. (**B**) SDS-PAGE analysis of the fractionated elution demonstrating the presence of the 75 kDa protein, kindlin-3 (labeled K3). Please click here to view a larger version of this figure.


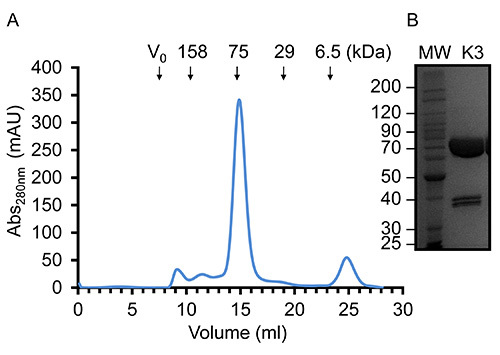
**Figure 4.** **Representative gel filtration chromatography and concentrated kindlin-3.** (**A**) Gel filtration elution profile of purified kindlin-3 using a Superdex S200 (16/60) in Tris-HCl, pH 7.5, 150 mM NaCl and 1 mM DTT at 20 °C. Based on the elution volume, kindlin-3 migrates as expected for a 75 kDa protein, suggesting that it is predominantly monomeric. (**B**) SDS-PAGE of highly concentrated purified recombinant kindlin-3 at 14.5 mg/ml. Please click here to view a larger version of this figure.


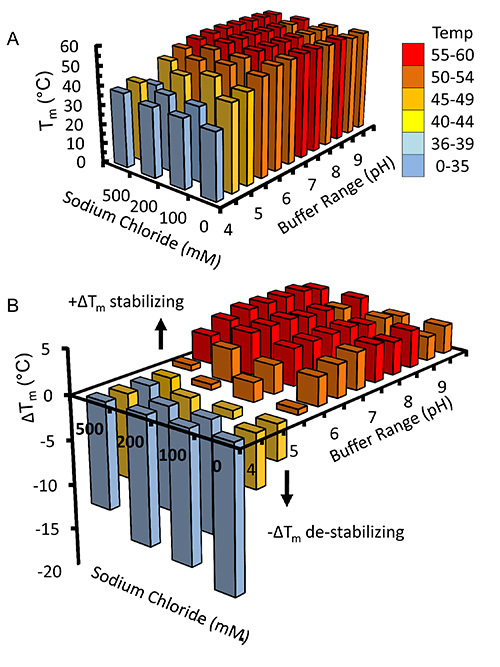
**Figure 5.** **Thermofluor-based Thermal Shift Assay for Buffer Screening.** Kindlin-3 was diluted into various buffers comprising a two-dimensional screen of pH versus sodium chloride concentration. Transition temperatures (temperature mid-points) were observed by fluorescence of the hydrophobically bound dye, Sypro orange (molecular probes) and calculated using the Opticon Monitor software. (**A**) A 3D histogram of the transition temperatures is plotted together with (**B**) the change in transition temperature from the calculated average of 50.4 °C. For clarity the bars are colored according to the range of temperatures to which they correspond. Please click here to view a larger version of this figure.

## Discussion

Baculovirus expression systems are becoming increasingly popular and an important tool for the production of milligram quantities of recombinant protein for protein characterization using biophysical studies, including X-ray crystallography. Despite being more experimentally demanding the baculovirus expression systems offer several advantages over *E. coli* one of which is a near-native environment for proteins of eukaryotic origin *e.g.* the presence of appropriate chaperones and opportunity for post-translational modification. In our own efforts to express kindlin-3, alternative expression hosts were used including mammalian cell-lines and bacterial expression strains (unpublished observations). Generally, the many *E. coli* strains tested produced very small quantities of recombinant kindlin-3 (~0.5 mg/L of culture; unpublished observations). However, the baculovirus-driven expression in insect cells was particularly effective and, in comparison to transient expression in mammalian cells, more amenable to generating the large biomass required for isolating a milligrams of recombinant cytoplasmic proteins (unpublished observations). We speculate that the presence eukaryotic chaperones may allow the efficient production of kindlin-3.

The baculoviridae infect insect cells and for recombinant protein expression the baculovirus used in this work is based upon the *Autographa californica *nuclear polyhedrosis virus (AcNPV). In nature the AcNPV, which infects the *Autographa californica *(alfalfa lopper) insect larvae, requires the polyhedrin protein to form occlusions whereby the virons are encapsulated in a crystalline protein matrix thus providing the necessary protection for their release. In cultured cells the formation of occlusion bodies is not required for replication and is therefore dispensable. In the case of expressing foreign proteins the polyhedrin protein gene can be replaced in a recombinant AcNPV with the gene for the protein of interest. AcNPV can infect other Lepidopteron species and for the purposes of recombinant protein expression army worm *Spodoptera frugiperda* pupal ovary cells are used. In the approach outlined here, the AcNPV bacmid (BAC10) is engineered so that an essential viral gene, ORF1629, is inactivated by the insertion of chloramphenicol acetyl transferase resulting in a knock-out bacmid (BAC10:KO_1629_), such that it is unable to form infectious baculovirions^28^. Cotransfection of Sf9 cells with linearized BAC10:KO_1629_ and the *FERMT3-*containing transfer vector repairs the inactive ORF1629, through transposition, resulting in a viable genome that has also incorporated the *FERMT3* gene under the control of the polyhedrin promoter^28^.

We describe a purification protocol for the isolation of highly pure recombinant mouse kindlin-3 via a three step chromatographic approach. The methods used here could easily be applied to other His-tagged proteins. We employed an ion exchange step to further purify kindlin-3 but we believe that this is a further pseudo-affinity step as kindlin-3 possesses a large number of basic residues, including a poly-lysine stretch within its F1 domain. Additionally, kindlin-3 is considered to bind to and interact with the cytoplasmic face of the plasma membrane, where it functions, and therefore we predict that the clustering of basic residues will allow the protein to counter the negatively charged membrane.

The buffers described in the purification protocols are considered standard and are frequently used in structural biology. The thermofluor assay (**Figure 5**) demonstrates that kindlin-3 is stable in most buffer conditions above pH 6.0. This was particularly useful and important for informing our experiments when studying kinldin-3:β1_A_ tail interaction by NMR, which yielded excellent spectra at pH 6.1 with low concentrations of NaCl^12^.

Before any biophysical study can be undertaken, it is important to demonstrate that the purified protein of interest is indeed correctly folded and is functionally active. In a previous publication we demonstrated that the recombinant kindlin-3 expressed and purified using this method was a monomer and monodispersed in solution, as assessed by size-exclusion chromatography, dynamic light scattering, analytical ultracentrifugation and small angle X-ray scattering, and was also capable of binding and recognizing the membrane-distal NPxY and upstream Serine/Threonine cluster of β_1A_ cytoplasmic tails^14^, thus confirming that it behaves like the native protein, which is in line with previous cellular and physiological studies^14,20,22^. The use of a thermal stability assay is an additional way of suggesting the proper folding of a protein of interest, as incorrectly folded protein will result in a high fluorescence background due to exposed hydrophobic residues.

The kindlin family of proteins has been the focus of much attention since their unexpected role as essential coactivators of integrins *in vivo *was discovered. This has triggered much effort to express them recombinantly and resolve their structures. To date limited success has been reported in expressing milligram quantities of full length recombinant protein but we have here described the use of a baculovirus system that permits large scale expression at levels where structural studies become feasible. By generating large quantities of recombinant kindlin-3 we anticipate that this will aid further studies of this protein. The baculovirus-driven method and purification workflow described here for recombinant murine kindlin-3 could also be used to express and purify the other kindlin isoforms, which are also difficult to express and also possess poly-lysine stretches, and may be further adapted for other cytoplasmic proteins, such as nucleic acid binding proteins, that fail to express in bacterial strains.

## Disclosures

The authors have nothing to disclose.
